# A cost-effective method to enhance adenoviral transduction of primary murine osteoblasts and bone marrow stromal cells

**DOI:** 10.1038/boneres.2016.21

**Published:** 2016-08-09

**Authors:** Atum M Buo, Mark S Williams, Jaclyn P Kerr, Joseph P Stains

**Affiliations:** 1 Department of Orthopaedics, University of Maryland School of Medicine, Baltimore, MD, USA; 2 Department of Microbiology and Immunology, University of Maryland School of Medicine, Baltimore, MD, USA

## Abstract

We report here a method for the use of poly-l-lysine (PLL) to markedly improve the adenoviral transduction efficiency of primary murine osteoblasts and bone marrow stromal cells (BMSCs) in culture and *in situ*, which are typically difficult to transduce. We show by fluorescence microscopy and flow cytometry that the addition of PLL to the viral-containing medium significantly increases the number of green fluorescence protein (GFP)-positive osteoblasts and BMSCs transduced with an enhanced GFP-expressing adenovirus. We also demonstrate that PLL can greatly enhance the adenoviral transduction of osteoblasts and osteocytes *in situ* in *ex vivo* tibia and calvaria, as well as in long bone fragments. In addition, we validate that PLL can improve routine adenoviral transduction studies by permitting the use of low multiplicities of infection to obtain the desired biologic effect. Ultimately, the use of PLL to facilitate adenoviral gene transfer in osteogenic cells can provide a cost-effective means of performing efficient gene transfer studies in the context of bone research.

## Introduction

The expression or silencing of specific genes via gene delivery strategies is an essential component of bone-related studies using primary osteogenic cells. Recombinant adenovirus vectors are routinely utilized for the *in vitro* transduction of primary osteoblasts and bone marrow stromal cells (BMSCs). However, obtaining suitable transduction efficiencies remains a persistent challenge, as successful adenoviral transduction of these cells requires very-high multiplicities of infection (MOI). This limits the utility and economics of adenoviral transduction. Furthermore, poor transduction efficiency can complicate data interpretation.

A viable option for improving adenoviral transduction efficiencies *in vitro* involves the use of polycationic compounds. When co-administered with virus particles, polycations are believed to neutralize repulsions between target cell membranes and viral particles, thereby improving viral attachment and transduction efficiency.^1–6^ Despite the potential impact of polycations, they are not routinely used in the bone field to enhance the adenoviral transduction of osteoblasts and BMSCs.

Here, we determined whether an inexpensive, common polycationic compound, poly-l-lysine (PLL), could enhance adenoviral-mediated gene transfer in osteoblasts and BMSCs. Previously, Orlicky and Schaack^7^ demonstrated that direct incubation of PLL with green fluorescent protein (GFP)-expressing adenoviral (Ad-GFP) particles results in near 100% transduction efficiency in 3T3-L1 preadipocyte cells that are normally difficult to transduce. Our data show that using PLL substantially enhances the adenoviral transduction efficiency of primary murine osteoblasts and BMSCs. Our method is accessible, requires only PLL and the adenovirus of interest, and a brief viral incubation period, yet results in marked improvements in transduction efficiency. We also demonstrate how this method can be exploited in knockdown studies to transduce floxed primary murine osteoblasts with Cre-expressing adenovirus at low MOI to obtain the expected biological effects.

## Materials and methods

### Animals

C57BL/6 wild-type and *Gja1*^*flox/flox*^ mice were purchased from the Jackson Laboratory (Bar Harbor, ME, USA) and maintained in the animal care facility at the University of Maryland School of Medicine. All animal studies were performed with approval by the Animal Care and Use Committee at the University of Maryland School of Medicine.

### Primary cell isolation and culture

Osteoblasts and BMSCs were isolated from mouse hind-limb long bones as previously described.^8,9^ Briefly, femurs and tibiae were dissected from 4-week-old C57BL/6 mice and *Gja1*^*flox/flox*^ mice. Under aseptic conditions, the epiphyses of the bone were removed, and the bone marrow flushed using complete medium (MEMα supplemented with 10% fetal bovine serum and 1% penicillin/streptomycin). For osteoblast preparation, flushed long bones were cut into small pieces and digested in collagenase A (Sigma, St Louis, MO, USA) solution for 2 h. Bone chips were then washed with complete medium and placed in a 100-mm tissue culture dishes (~20–25 chips per dish). Bone chips were kept at 37 °C and 5% CO_2_ in a humidified incubator and were fed with fresh complete medium every 3 days. Cells were maintained for 2 weeks or until the culture reached about 70% confluence. For BMSCs preparation, the combined marrow suspension was filtered using a 70-μm cell strainer. Cells were seeded into a 100-mm tissue culture dish and incubated at 37 °C and 5% CO_2_ for 2 days. On the second day, the monolayer was washed with Hank’s Balanced Salt Solution to remove non-adherent cells. Adherent cells were grown and maintained for 7–10 days until 70% confluence. All primary cell cultures were fed every 3 days with fresh complete medium.

### Adenoviral constructs and amplification

The enhanced Ad-GFP and the GFP-tagged Cre recombinase adenovirus (Ad-Cre), were both purchased from Vector Biolabs (Philadelphia, PA, USA) at a viral titer of 1×10^10^ plaque-forming units per mL (PFU·mL^−1^). Ad-GFP expresses enhanced GFP under the control of a cytomegalovirus (CMV) promoter, whereas Ad-Cre contains GFP downstream of an IRES sequence. Viruses were amplified by infecting overnight cultures of 293a packaging cells (Life Technologies, Eugene, OR, USA) seeded in complete DMEM at a cell density of 54 500 cells per cm^2^ (or 3×10^6^ cells in a 100-mm tissue culture dish) with 1 μL of each virus in separate dishes. Once 70%–80% of the packaging cells were floating in the medium (~3–5 days) the suspended cells were harvested, and the virus particles released by three repeated freeze-thaw cycles and aliquotted into sterile cryovials. Titers of amplified virus were determined by conducting an agarose plaque assay on serial dilutions of the viral stocks (Life Technologies).

### Adenoviral transduction

Primary osteoblasts and BMSCs were transduced the day after target cells were seeded into multi-well tissue culture plates with or without cell culture-grade PLL in order to assess its impact on adenoviral transduction. Serum-free MEMα was prepared at 50% of the typical well culture volume (for example, 0.5 mL in a 12-well plate). For the PLL-mediated transduction condition, cell culture-grade PLL (Sigma; 0.5 μg·mL^−1^) was added prior to the addition of virus. The tubes were allowed to incubate for 5–10 min at room temperature. The virus-containing medium was then used to replace the culture media already on cells, and the cells were returned to the incubator. After a transduction period of 1 h, the viral medium was discarded and replaced with fresh complete medium. All assays were conducted 3 days post transduction to allow for optimal expression.

### Fluorescent microscopy and flow cytometry

Cultured primary osteoblasts were washed with Hank’s Balanced Salt Solution, trypsinized and seeded into 12-well plates at a seeding density of 80 000 cells per well and transduced with Ad-GFP at the MOIs specified above. After 72 h, cells were assessed visibly by fluorescent microscopy. DiI (Life Technologies) was used to label all cells in the dish according to manufacturer’s directions. Cells were imaged on a Nikon inverted fluorescence microscope (Nikon, Tokyo, Japan).

For flow cytometry, transduced cells were spun down in a microcentrifuge to remove the supernatant, and the pelleted cells were washed with ice-cold Hank’s Balanced Salt Solution and then resuspended in 500 μL Hank’s Balanced Salt Solution containing 5 μg·mL^−1^ propidium iodide, transferred to fluorescence-activated cell sorting tubes and kept on ice before being processed for flow cytometry as described.^10^

### Quantitative PCR

Total RNA was isolated from transduced cells using Tripure reagent (Roche, Indianapolis, IN, USA). Reverse transcription quantitative PCR was carried out, and data are shown relative to the expression of *Gapdh*, *Rpl13*, and *Hprt* using geNorm v3.5 software (Ghent University Hospital, Ghent, Belgium), as described previously.^11^ The primer sets for *Gapdh*, *Rpl13*, *Hprt*, *Gja1*/Cx43, *Bglap*/osteocalcin, *osx*/Osterix, and *Runx2* are described previously.^11,12^

### Western blotting

Western blots were performed as previously described.^13^ Briefly, sample were subjected to separation on 10% SDS–PAGE gels and then transferred to polyvinylidene difluoride membranes (Millipore, Bedford, MA, USA). Membranes were blocked in 5% non-fat dry milk, and were incubated with primary antibodies followed by horseradish peroxidase-conjugated secondary antibodies. Membranes were visualized using Clarity ECL western blotting substrate (Bio-Rad, Hercules, CA, USA), and imaged with a UVP EpiChem gel documentation system (UVP Bioimaging Systems, Upland, CA, USA). The rabbit anti-Cx43 antibody was purchased from Sigma. The mouse anti-GAPDH and rabbit anti-osterix antibodies were purchased from Millipore.

### *In situ* transduction of bone samples

Long bones and calvaria were isolated from *Gja1*^*flox/flox*^ mice and were cleaned of their soft tissue, flushed of their marrow and subjected to collagenase A digestion as described above. Each individual calvaria was digested twice with 30-min rounds of collagenase A, followed by a 30-min incubation with Trypsin EDTA, and finally another 30-min incubation with collagenase A. Digested long bones and calvaria were each placed in separate 12-well plates and cultured overnight in complete medium. The following day, the whole-bone samples were infected with Ad-GFP at a viral titer of 2×10^7^ PFU·mL^−1^ in the presence or absence of PLL and were analyzed using fluorescent microscopy 3 days later.

For viral transduction of bone fragments, long bone samples were dissected into smaller pieces prior to transduction with PLL-coated Ad-Cre, whereas calvaria were left intact. Transduced long bone fragments and calvaria were collected into RIPA lysis buffer 3 days later and were subjected to tissue homogenization using metallic beads and a Qiagen Tissuelyser LT (Qiagen, Hilden, Germany) to grind up the bone into dust. Protein extracts from these homogenates were processed for Cx43 immunoblotting as described above.

### Cell viability assay

Cell viability of Ad-Cre-transduced osteoblasts was assessed with the Cell Counting Kit-8 colorimetric assay and used according to the manufacturer’s instructions (Dojindo, Rockville, MD, USA). The absorbance of the culture medium was measured at 450 nm using a microplate meter. Data are plotted as blank corrected optical densities.

### Statistical analysis

Unless indicated, experiments were performed in triplicate wells and repeated at least three times. Flow cytometry experiments were performed twice, and data figures present results from a representative experiment. Histograms show means±s.d. Data were assessed for statistical significance by analysis of variance followed by a Dunnet’s *post hoc* test using GraphPad Prism (v6) (GraphPad Software, La Jolla, CA, USA). *P*<0.05 are considered statistically significant.

## Results

### PLL enhances transduction efficiency of a GFP-expressing adenovirus in primary osteoblasts and BMSCs

**Figure 1:**
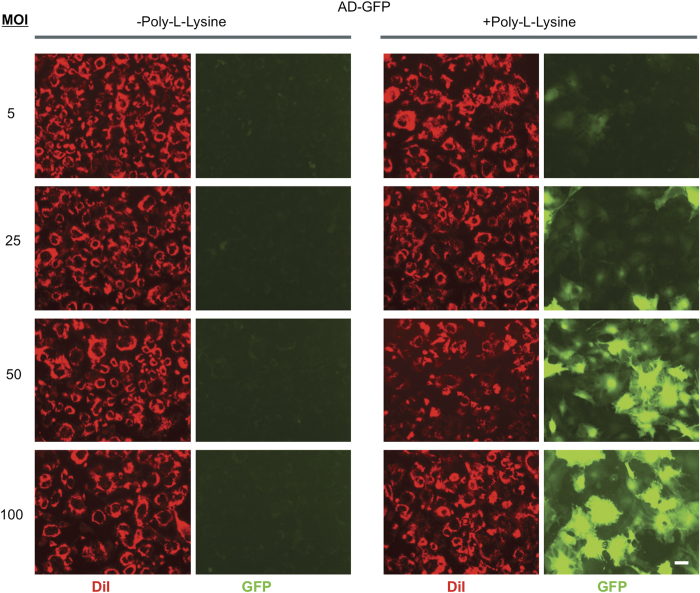
PLL increases the number and intensity of green fluorescent cells *in vitro*. Primary murine osteoblasts were cultured and transduced with a GFP-encoding adenovirus at the indicated MOIs in the presence or absence of PLL. Cells were labeled with DiI (red) and fluorescence was detected by fluorescence microscopy. As negative controls, sets of cells were cultured with or without PLL but were not transduced with adenovirus. Data are from a representative experiment. Scale bar=20 μm. GFP, green fluorescence protein; MOI, multiplicities of infection; PLL, poly-l-lysine.

We sought to obtain evidence that PLL enhances adenoviral transduction efficiency in primary osteoblasts and BMSCs. To establish this, we first evaluated the effect of PLL on the ability of a Ad-GFP to infect primary osteoblasts. Primary osteoblasts were isolated from C57BL/6 mice and were transduced with viral medium containing Ad-GFP at MOIs of 5, 25, 50, and 100 PFU per cell, in the absence or the presence of 0.5 μg·mL^−1^ PLL. Cells were assayed using fluorescent microscopy 72 h post transduction to qualitatively assess transduction efficiency. The number of primary osteoblasts expressing GFP was remarkably higher when cells were treated with PLL at every MOI tested (Figure 1). Strikingly, little fluorescence was detected at any MOI when cells were transduced in the absence of PLL. Similar results were observed in BMSCs (data not shown).

**Figure 2:**
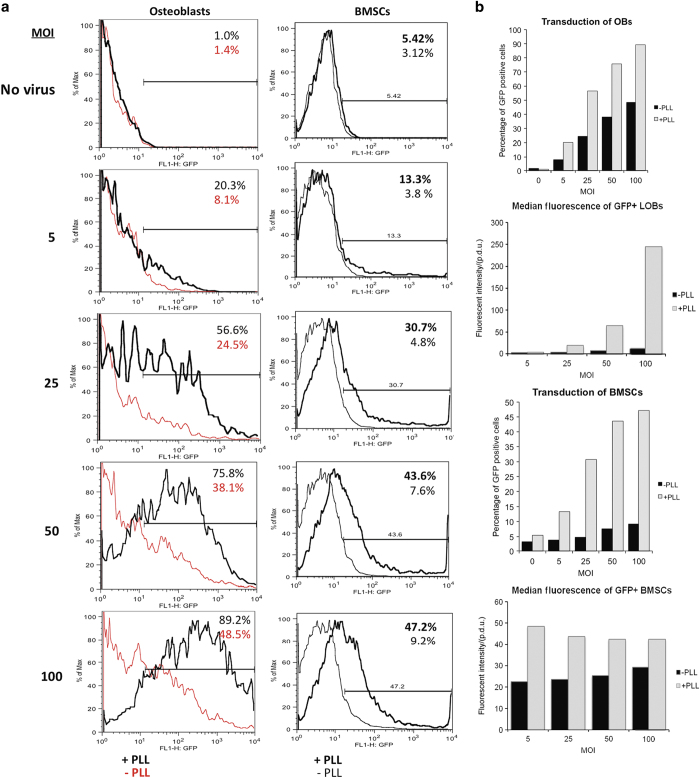
PLL markedly enhances adenoviral transduction of osteoblasts and BMSCs *in vitro*. (**a**) Flow cytometry was performed on primary murine osteoblasts and BMSCs that had been transduced with a GFP-encoding adenovirus at the indicated MOIs in the presence or absence of PLL. The percentage of GFP+ cells is indicated for each condition. Quantitation of the percentage of GFP-positive cells for osteoblasts (OBs) and BMSCs at each MOI is shown in the histogram. (**b**) The median fluorescent intensity of the GFP+ population of long bone osteoblasts (LOBs) and BMSCs is shown. Data are from a representative experiment. BMSC, bone marrow stromal cell; GFP, green fluorescence protein; MOI, multiplicities of infection; PLL, poly-l-lysine.

We then performed flow cytometry to quantitate the percentage yield of GFP-positive (GFP+) cells. Consistent with the fluorescence microscopy data, flow cytometry revealed that PLL increased both the fluorescence intensity and percentage of GFP+ cells for both osteoblasts and BMSCs at every MOI (Figure 2). For the osteoblast transductions, when PLL was present, an MOI of 100 resulted in 89.2% GFP+ cells versus a yield of 48.5% GFP+ cells when PLL was not (Figure 2a). In addition to an increase in the percentage of GFP+ cells, the median fluorescence intensity is notably higher in cultures in which the cells were transduced in the presence of PLL (Figure 2b). For the BMSC transductions, PLL was more effective at enhancing the number of GFP+ cells, resulting in an average fold increase in transduction efficiency of 5.13 compared with cells transduced in the absence of PLL (Figure 2a). However, the percentage yield of GFP+ cells only reached 47.2% at an MOI of 100 even in the presence of PLL. Furthermore, the inclusion of PLL resulted in a ~2-fold increase in median fluorescent intensity, but, unlike primary osteoblasts, the median fluorescence was not dose-dependently increased by the MOI (Figure 2b), perhaps underscoring the relative difficulty of transducing BMSCs with adenovirus. Taken together, these results show that PLL markedly increases the number of transduced osteoblasts and BMSCs as well produces more robust GFP expression in these transduced cells than cells transduced without PLL. Thus, PLL permits robust transduction at much lower MOIs than are achieved in the absence of PLL.

### PLL does not impact cell viability and osteoblast differentiation

**Figure 3:**
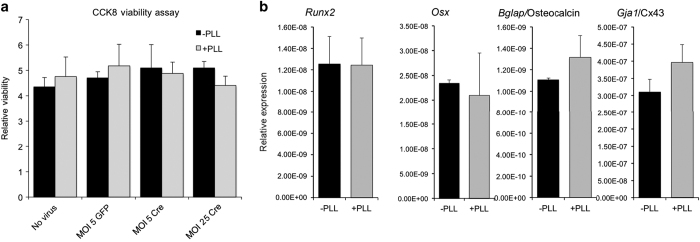
Adenoviral transduction with PLL does not affect cell viability or osteoblast differentiation. (**a**) The viability of primary osteoblasts was assessed with a colorimetric CCK-8 cell viability assay at the indicated MOIs in the presence or absence of PLL. Cells were transduced with GFP (negative control) or Cre recombinase expressing adenovirus as indicated. Viability was assessed 72 h post transduction. Pathlength corrected OD450 values are shown as mean±s.d. No statistical significance between samples was observed. (**b**) mRNA levels of osteoblast genes were determined by quantitative RT-PCR from cells transduced with Ad-GFP (MOI=5) at the presence or absence of PLL and then cultured in osteogenic media for 7 days. No statistical significance between samples was observed. CCK-8, Cell Counting Kit-8; GFP, green fluorescence protein; MOI, multiplicities of infection; PLL, poly-l-lysine; RT-PCR, reverse transcription–PCR.

We then wanted to verify that PLL did not adversely impact cell viability. In addition, we wanted to ensure that the substantial increases in adenoviral particle infectivity and exogenous gene expression did not affect cell viability, as proteins like Cre are known to cause cell toxicity.^14^ We conducted a colorimetric Cell Counting Kit-8 viability assay on osteoblasts that were transduced with Ad-Cre in the presence or the absence of PLL at MOIs of 5 and 25. As a negative control, PLL was added to cells alone without virus, which had no negative impact on cell viability (Figure 3a). We observed that PLL did not significantly reduce cell viability at any of the MOIs tested. Similarly, the expression of genes associated with osteoblast differentiation was unaffected in PLL-treated samples relative to controls (Figure 3b).

### PLL generates efficient knockdown of LoxP-flanked genes facilitated by a Cre-expressing adenovirus at low MOI

We then wanted to determine the functional significances of enhancing adenoviral transduction efficiency with PLL. For these studies, we used osteoblasts from *Gja1*^*flox/flox*^ mice. *Gja1* is the gene name for the gap junction protein Cx43, an important regulator of osteoblast differentiation.^15^ Furthermore, deletion of Cx43 in osteoblast lineage cells has a broadly reported impact on several osteogenic genes, including *Runx2*, *Osx*/Osterix, and *Bglap*/osteocalcin.^16,17^ Thus we could determine whether inclusion of PLL in the transduction media at such MOIs could still generate the anticipated biological consequence. Accordingly, primary cells were transduced with Ad-Cre at the indicated MOIs in the absence or the presence of PLL. A set of cells was also transduced with Ad-GFP at an MOI of 5 as a negative control and to serve as a baseline for comparison.

**Figure 4:**
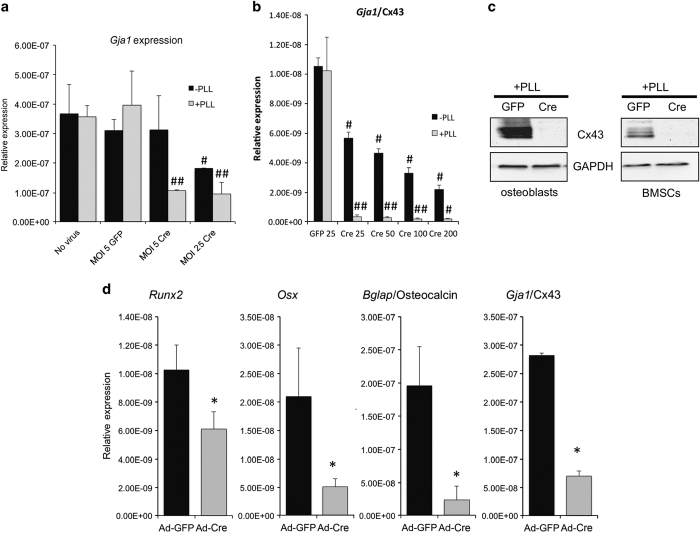
PLL both improves the efficiency of adenoviral-Cre-mediated deletion of a floxed allele in primary osteoblasts and BMSCs from *Gja1*^*fl/fl*^ mice and recapitulates the expected biological responses to *Cx43* gene deletion at low MOI. Quantitative real time RT-PCR of the expression of the *Gja1* allele in (**a**) primary mouse osteoblasts or (**b**) primary mouse BMSCs 72 h post transduction with a Cre recombinase-encoding adenovirus (Cre) or a negative control GFP-encoding adenovirus (Ad-GFP) at the indicated MOIs in the presence or the absence of PLL. (**c**). Westerns blot of Cx43 expression in whole cell extracts from primary osteoblasts or BMSCs from *Gja1*^*fl/fl*^ mice that had been transduced with GFP (negative control) or Cre recombinase expressing adenovirus (MOI=5, respectively) in the presence of PLL. The multiple bands observed are due to the multiple phosphorylation states of Cx43. (**d**) Quantitative real time RT-PCR of the expression of osteoblasts genes in BMSCs differentiated for 7 days post transduction with GFP (negative control) or Cre recombinase expressing adenovirus (MOI=5) in the presence of PLL. All histograms represents means±s.d. and *P<0.05. #, indicates *P*<0.05 relative to the GFP transduced control; ##, indicates *P*<0.05 relative to the GFP transduced control and the corresponding –PLL sample. GFP, green fluorescence protein; MOI, multiplicities of infection; PLL, poly-l-lysine; RT-PCR, reverse transcription–PCR.

Addition of PLL dramatically enhanced the Cre-mediated deletion of the *Gja1* gene in long bone osteoblasts isolated from *Gja1*^*flox/flox*^ mice, resulting in decreased *Gja1* mRNA and Cx43 protein by quantitative reverse transcription–PCR and western blotting (Figure 4a; Supplementary Figure 1). At an MOI of 5, we observed a 73% reduction in *Gja1* mRNA in osteoblasts transduced with PLL, whereas there was no reduction in *Gja1* mRNA in the absence of PLL at this MOI (Figure 4a). PLL-induced deletion of *Gja1* was not further enhanced at an MOI of 25, although we saw a 41% reduction in *Gja1* mRNA when osteoblasts were transduced with Ad-Cre only. This signifies that transduction of Ad-Cre and deletion of *Gja1* is at least eight times more effective when PLL is present. Analogous results demonstrating the enhanced Cre-mediated deletion of Cx43 at low MOIs in the presence of PLL were obtained in BMSCs (Figure 4b). Using PLL resulted in efficient Cre-mediated deletion of Cx43 in both osteoblasts and BMSCs even at low MOI (Figure 4c) and decreased the expression of osteoblast genes that are affected by Cx43 deletion (Figure 4d). Thus, these data show that PLL can be used to improve transduction of Ad-Cre in primary osteoblasts and BMSCs and result in efficient deletion of floxed genes even at low MOIs. Furthermore, in the presence of PLL, low MOI transduction of *Gja1*^*flox/flox*^ BMSCs with Ad-Cre generated the expected downregulation of osteoblast gene expression, consistent with effective gene deletion.

### Bone cells *in situ* can be successfully transduced with PLL

**Figure 5:**
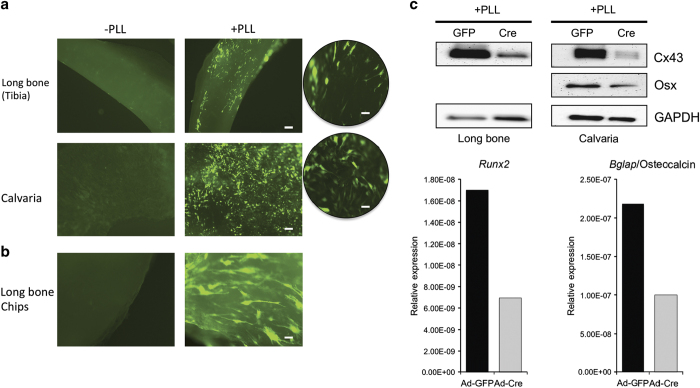
PLL can be used to drastically enhance transduction of osteogenic cells *in situ*. (**a**) Fluorescent images of intact tibias and calvaria transduced with Ad-GFP in the absence (left side) or presence (right side) of PLL. Scale bar equals 100 μm. A higher magnification view (circles) is also displayed. Scale bar equals 20 μm (**b**) Smaller long bone fragments transduced with Ad-GFP in the absence (left) or presence (right) of PLL imaged. Scale bar equals 20 μm. (**c**) Western blot probing for Cx43, Osterix (calvaria only) and GAPDH using tissue homogenates obtained from long bone pieces and calvaria transduced with Ad-GFP and Ad-Cre in the presence of PLL only. Quantitative real time RT-PCR of the mRNA levels of *Runx2* and *Bglap*/osteocalcin from the adenoviral transduced calvaria shown in (**a**). GFP, green fluorescence protein; MOI, multiplicities of infection; PLL, poly-l-lysine; RT-PCR, reverse transcription–PCR.

Adenoviral transduction has great potential *in vivo* and *ex vivo.* Although advances in vector design are continually being made to assist in these applications,^18^ we were curious whether the addition of PLL to the adenoviral transduction medium is potent enough to treat whole bone samples *ex vivo* and infect cells *in situ.* To test this, femurs, tibias, and calvaria from *Gja1*^*flox/flox*^ mice were flushed and cleaned before transduction with Ad-GFP either in the presence or absence of PLL. As we could not ascertain the number of cells within each sample of bone, we utilized an arbitrarily determined titer of 2x10^7^ PFU·mL^−1^. Examination of intact tibia or calvaria cultured *ex vivo* (Figure 5a) or dissected long bone fragments in culture (Figure 5b) via fluorescent microscopy revealed that whole bones transduced with PLL displayed a greater number of GFP-fluorescing cells than samples transduced without PLL. To assess the functional implications of this, we also examined whole-cell extracts from *Gja1*^*flox/flox*^ long bone and calvaria samples that were transduced with Ad-Cre in the presence of PLL in order to determine whether PLL could facilitate efficient downregulation of Cx43 protein *in situ*. We observed a reduction in Cx43 protein expression in long bones and calvaria that were co-transduced with Ad-Cre and PLL (Figure 5C). As we observed in primary cell culture, Cre-mediated deletion of Cx43 in calvarial explants at these low MOIs reduced Osterix protein expression and the mRNA levels of *Runx2* and *Bglap*/osteocalcin genes, consistent with the expected biologic response of Cx43 deletion (Figure 5C). These data indicate that PLL can be used to enhance the direct transduction of bone cells *in situ*, and even at these low MOIs we achieve the expected biologic response.

## Discussion

Optimizing adenoviral transduction in bone would be beneficial for *in vitro*, *in vivo*, and *ex vivo* applications requiring exogenous gene expression. Adenoviruses are particularly appealing gene delivery vectors because they can be engineered to be replication incompetent, have a low risk of random integration into the host genome, and, although transient, can offer prolonged expression of the exogenous material, up to 90 days in some instances.^19^ However, some cell types are harder to infect with adenovirus than others, which presents a major limitation. Primary osteoblasts and BMSCs are particularly difficult to transduce, in part due to low levels of Coxsackievirus and Adenovirus Receptor expression in mesenchymal-derived cells.^20^ Studies utilizing adenoviral vectors to infect osteogenic cells therefore employ transduction protocols that are highly inefficient and require extremely high viral titers in order to achieve a high enough percentage of infected cells. The range of MOIs typically hovers around 100–200 PFU per cell, although studies have reported MOIs as high as 800 PFU per cell.^21^ Some conventional transduction protocols circumvent this limitation by co-administering viral particles with proprietary polycationic-containing reagents, although the cost of these reagents may be prohibitive for frequent transduction experiments with a high number of wells.

Here, we implement fluorescent microscopy, fluorescence-activated cell sorting analysis, and Cre-mediated *Gja1*-floxed knockdown approaches and show that PLL can be used to effectively improve adenoviral transduction of murine primary osteoblast and BMSCs with no loss in cell viability and no overt impact on osteoblast differentiation. In both cell types, we observed that PLL increased both the percentage of cells transduced and increased the efficiency of the transduction per cell, as shown by increased median fluorescence per cell in Ad-GFP transduced cells and our immunofluorescence data. We also show that despite, the use of low MOIs in the PLL treated samples, we observe efficient Cre-mediated gene deletion of Cx43 resulting in the expected biological response (that is, the downregulation of several osteoblast genes). Our data support the notion that, not only PLL can enhance the adenoviral transduction in these cells permitting the use of low MOIs, but these low MOI transductions remain efficacious in generating the expected biological response.

We observed these effects at a PLL concentration of 0.5 μg·mL^−1^, based on a preliminary dose response curve (data not shown). Our dose is consistent with a prior study by Orlicky and Schaack^7^ demonstrating that in 3T3-L1 preadipocytes, transduction efficiency peaks at 0.5–1.0 μg·mL^−1^ PLL but starts to decrease at higher concentrations. In contrast to the long incubation times needed to transduce 3T3-L1,^7^ we observed efficient transduction at low MOIs with much shorter incubation times with both PLL and viral particles, further underscoring the practical utility of this method for enhancing adenoviral transduction in these cells. This method is broadly applicable to osteogenic cells, as we have used it to routinely adenoviral transduce calvarial osteoblasts in addition to the cells presented here. We do not include these data as we have not quantitatively evaluated +PLL versus −PLL conditions in these cells; however, our effective MOIs are much lower than they were prior to using PLL.

Other studies have examined the efficacy of polycations in enhancing adenoviral transduction. Although polycationic compounds such as protamine, DEAE-dextran, polybrene, PLL, GL-67, and polyethylene glycol have been reported to improve adenoviral transduction efficiency in a variety of cell types,^1–3,5,22,23^ the bone field does not routinely utilize these types of reagents to enhance their adenoviral transduction experiments. One of the few studies looking the effects of polylysine-enhanced adenoviral transduction in BMSCs did so by covalently attaching a series of lysine residues to the capsid fiber of the adenovirus vector.^20^ Our method is more straightforward and thus more accessible. Although we do not attempt to directly compare the efficacy of PLL to other polycationic reagents in osteogenic cells, the utility of our findings is that PLL provides an easy, cost-effective option to rapidly improve adenoviral transduction of osteogenic cells without having to manipulate the adenoviral structure.

We also found that PLL could be implemented to augment the direct transduction of bone specimens and enhance gene delivery to cells *in situ*. Confocal microscopic images of these bone chips also display the sizeable number of green fluorescing cells resulting from PLL-mediated transduction (Supplementary Figure 2), suggesting that both high-resolution studies of protein localization and live imaging of reporter proteins within live bone chips can be achieved with this method. Importantly, we demonstrate functional efficacy of this *in situ* transduction method, as we observed downregulation of Cx43 protein expression, as well as the concomitant reduction of osteoblast markers known to be affected by loss of Cx43,^16,17^ when bones from *Gja1*-floxed mice were directly transduced with Ad-Cre *ex vivo*. Although promising, these findings suggest that only cells near the surface of the bone fragments are transduced (Supplementary Figure 2b). Nevertheless, the possibility of conducting gene delivery experiments *in* *situ* while keeping the structure of the bone relatively intact seems to be attainable with PLL and also opens up the potential of other applications involving *in situ* transduction.

In conclusion, we show for the first time that PLL can be used to safely and effectively improve adenoviral transduction of murine primary osteoblast and BMSCs. We demonstrate that the addition of a small volume of PLL to a final concentration of 0.5 μg·mL^−1^ in cell culture medium can result in a twofold and fivefold increase in transduction efficiency of primary osteoblasts and BMSCs, respectively, with GFP-expressing adenovirus. We also evaluated the efficacy of PLL in Cre-mediated knockdown strategies and discovered that transducing primary cells from *Gja1*-floxed mice with Ad-Cre using the same concentration of PLL is at least five times more effective at knocking down *Gja1* mRNA and Cx43 protein than just viral media without PLL. Thus, we envision that the greater impact of the effectiveness of PLL-enhanced adenoviral transduction efficiency is that it provides investigators with a rapid and cost-effective option to alter gene expression in primary osteoblasts or BMSCs.
