# Supplementary figures and images for: A cost-effective method to enhance adenoviral transduction of primary murine osteoblasts and bone marrow stromal cells
Source: Bone Res. 2016 Aug 9;4:16021–. doi: 10.1038/boneres.2016.21 (PMC4977485; doi:10.1038/boneres.2016.21)

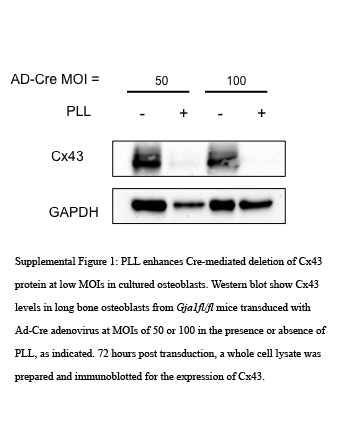

Supplement: Supplementary Figure S1 [file boneres201621-s1.tiff]

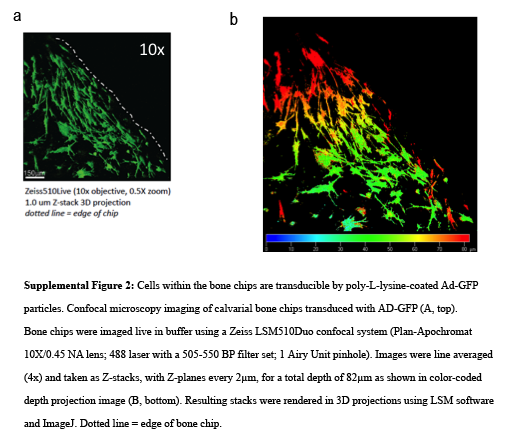

Supplement: Supplementary Figure S2 [file boneres201621-s2.tiff]
